# P17 Vancomycin therapeutic drug monitoring—a call for change?

**DOI:** 10.1093/jacamr/dlac004.016

**Published:** 2022-02-16

**Authors:** Lara Payne, Christian J. Buckingham, Paul Wade, Aisling F. Brown

**Affiliations:** Department of Infectious Diseases, Guy’s and St Thomas’ NHS Foundation Trust, London, UK; Department of Infectious Diseases, Guy’s and St Thomas’ NHS Foundation Trust, London, UK; Department of Infectious Diseases, Guy’s and St Thomas’ NHS Foundation Trust, London, UK; Department of Infectious Diseases, Guy’s and St Thomas’ NHS Foundation Trust, London, UK

## Abstract

**Objectives:**

Despite the widespread use of vancomycin, optimization of dosing can be difficult. Increased vancomycin concentrations are associated with nephrotoxicity, while subtherapeutic levels may lead to inadequate antibacterial therapy and risk development of antimicrobial resistance. Recent guidelines have recommended the use of loading doses and switching to AUC_24_ TDM, targeting an AUC_24_:MIC ratio of 400–600 μg·h/mL to minimize toxicity while maintaining similar effectiveness for the treatment of serious infections. We aimed to review adherence to current local vancomycin dosing and conventional trough monitoring guidelines, as well as record time to, and factors related to, achieving target vancomycin troughs.

**Methods:**

Fifty consecutive electronic prescriptions were screened for IV vancomycin use in treatment of active infections in a single acute Trust during April/May 2021. These were cross-referenced with microbiological, clinical and biochemical data from patient records. Variables were analysed by parametric or non-parametric methods on the basis of their normality distribution using GraphPad Prism.

**Results:**

Forty-one patients were included of which 39 had a vancomycin level measured. Mean age was 57 years (range 19–88) and BMI 28 (range 17–44). Nineteen had unstable renal function, including one receiving intermittent haemodialysis. Median CL_CR_ was 95  mL/min (IQR 66–129). Indications were included on the prescription for 26. Median duration of vancomycin was 4 days (range 2–12), and half of patients ultimately switched therapy to an alternate agent . Target troughs were documented in 29 (70%), 9 of which had a target trough ≥15 μg/mL. Twenty-four had positive microbiology samples, 19 of which were organisms against which vancomycin would have expected activity. Five (13%) failed to achieve trough levels >10, 6/39 (15%) achieved troughs of 10–15, 13/39 (33%) 15–20, and 15/39 (38%) had troughs of >20 μg/mL (Figure 1). Where it was achieved, the median time to level ≥10 μg/mL was 46 h (range 11–202) and time to level ≥15 μg/mL was 68 h (range 18–243). Twenty-five (60%) patients required titration above the currently recommended initial dose, and 22 required doses of 1500 mg twice daily or higher. BMI did not correlate with vancomycin level achieved.

**Conclusions:**

A significant proportion of patients with serious infections fail to achieve or have a considerable delay in achieving therapeutic vancomycin trough. An even greater proportion have troughs above target and are at risk of nephrotoxicity. These findings support a potential switch to more personalized regimens using loading doses and AUC_24_ monitoring where vancomycin is the drug of choice. This would complement other stewardship measures to limit overall vancomycin use, achieve earlier and more complete source control, and optimize use of alternative antimicrobial therapies with more predictable PK/PD.

**Figure 1.**
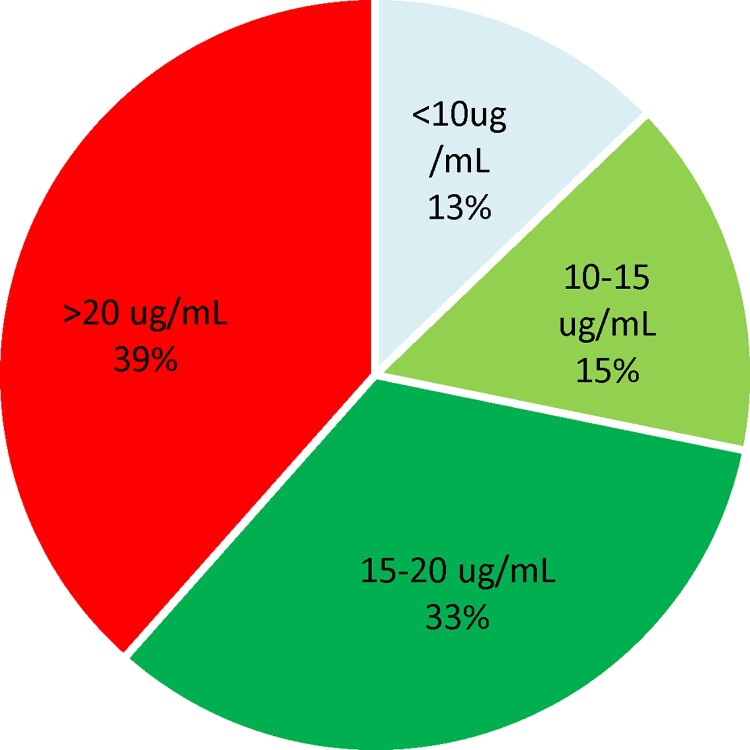
Vancomycin trough ranges.

